# Risk prediction model of early vascular aging based on nomogram in patients with type 2 diabetes mellitus: a cross-sectional study in a Chinese population

**DOI:** 10.3389/fmed.2025.1541259

**Published:** 2025-09-30

**Authors:** Xin Zhao, Jianbin Sun, Sixu Xin, Xiaomei Zhang

**Affiliations:** Department of Endocrinology, Peking University International Hospital, Beijing, China

**Keywords:** type 2 diabetes mellitus, early vascular aging, nomograms, pulse wave velocity, receiver operating characteristic

## Abstract

**Background:**

Early Vascular Aging (EVA) is a significant risk factor for cardiovascular disease in patients with type 2 diabetes mellitus (T2DM). This study aimed to explore the risk factors for EVA in patients with T2DM in China and develop nomograms for EVA in patients with T2DM.

**Methods:**

We retrospectively analyzed data from 1,543 patients with T2DM. The patients were divided into non-EVA and EVA groups based on ankle-brachial pulse wave velocity (PWV).

**Results:**

(1) The risk factors for EVA in male included longer diabetic duration (OR = 1.09 95 CI% 1.06–1.11), high blood pressure (OR = 2.06, 95 CI% 1.51–2.81), smoking (OR = 1.96 95 CI% 1.17–3.27), diabetic nephropathy (DN; OR = 1.60 95 CI% 1.10–2.32), and diabetic retinopathy (DR; OR = 2.93 95 CI% 2.00–4.29). The risk factors for EVA in females included longer duration of diabetes (OR = 1.04 95 CI% 1.01–1.07), smoking (OR = 2.02, 95 CI% 1.13, 3.59), high blood pressure (OR = 1.91, 95 CI% 1.22–2.79), diabetic nephropathy (OR = 1.61 95 CI% 1.02–2.52), and diabetic retinopathy (OR = 3.61 95 CI% 2.24–5.74). (2) The results showed that the nomogram-based risk prediction model achieved an area under the curve of 0.73 for men and 0.74 for women. The overall predictive accuracy of the nomogram for EVA in men was 67.85%, and its specificity and sensitivity were 73.74 and 62.33%, respectively. The overall predictive accuracy of the nomogram for EVA in females was 69.29%, and its specificity and sensitivity were 66.55 and 71.93%, respectively.

**Conclusions:**

The nomogram-based risk prediction model for EVA in T2DM patients showed good discriminative ability and predictive accuracy. It provides clinicians with a reliable tool to estimate the risk of EVA in T2DM patients, allowing for early interventions and reduction of cardiovascular diseases in high-risk populations.

## 1 Introduction

Vascular aging is an age-related degenerative disease of the cardiovascular system that occurs with age. It is often accompanied by elastic arterial stiffness, decreased compliance, weakened vascular endothelial cell (VEC) function, vascular repair, and neogenesis. Vascular aging can be classified into physiological and pathological types.

The former is physiological degeneration that occurs after maturity, and the latter is mostly caused by diseases such as type 2 diabetes mellitus (T2DM) and atherosclerosis. Both diseases significantly increase the risk of cardiovascular diseases (1). Early vascular aging (EVA) refers to premature vascular aging, in which the actual vascular age differs from the biological vascular age. The vascular state is an accelerated vascular aging state that cannot be explained by aging. EVA is characterized by vascular stiffness, endothelial dysfunction, impaired vasodilation, and chronic inflammation. The pulse wave velocity (PWV) is commonly used to evaluate the degree of vascular sclerosis in patients with diabetes. The diagnostic criterion for EVA is a PWV value, after adjusting for age, that is two standard deviations higher than the normal reference value of the healthy population (2).

T2DM is associated with early onset vascular senescence, which can damage the function of intimal vascular endothelial cells (VEC) and vascular adventitial fibroblasts (VAFs), and cause vascular aging (3). Most of these injuries are related to glucose and lipid homeostasis, inflammatory reactions, oxidative stress, autophagy pathways, angiotensin II (Ang II), and other factors.

Our previous study showed that with increasing age, the degree of vascular sclerosis, blood pressure, blood glucose, and high blood lipid levels can accelerate the speed of vascular sclerosis and cause changes in the vascular physiological state earlier than their physiological age (4). Maintaining normal vascular aging is crucial for vascular health and delaying the occurrence of cardiovascular diseases, which can be prevented and treated by improving lifestyle or drug use, thereby increasing the incidence and mortality of vascular-related diseases (5). EVA is a significant risk factor for cardiovascular disease in patients with T2DM. Accelerated vascular aging process in T2DM patients contributes to increased morbidity and mortality due to cardiovascular events. Therefore, establishing an accurate risk prediction and simple-to-use model for EVA is crucial for identifying high-risk patients and implementing early interventions.

Currently, there are few studies on the relationship between T2DM and EVA in China, and no studies have identified the risk factors for EVA and established related prediction models in T2DM patients.

This study explored the incidence and risk factors of EVA in patients with T2DM and established a nomogram-based model according to the related risk factors to provide new evidence for the early identification and intervention of EVA in patients with T2DM.

## 2 Materials and methods

We followed the methods described in our study before (6).

### 2.1 Ethics statement

This study has been approved by the Ethics Committee of Peking University International Hospital. The ethical approval number for this study is 2021-KY-0051-02.

### 2.2 Research subjects

This study retrospectively included 1,823 T2DM patients from January 2017 to August 2021 at Peking University International Hospital. According to the inclusion and exclusion criteria, 1,523 patients, including 983 males and 560 females, were enrolled in the study, with an average age of 55.59 ± 13.12 years.

All patients met the diagnostic criteria for diabetes set by the World Health Organization (WHO) in 1999 (7). The exclusion criteria were as follows: (1) patients with type 1 diabetes, gestational diabetes, and other special types of diabetes; (2) suffering from diseases such as parathyroid dysfunction, thyroid dysfunction, and adrenal dysfunction; (3) suffering from severe liver and kidney dysfunction; and (4) suffering from atherosclerosis, malignant tumor, and blood system disease.

### 2.3 General conditions and clinical data

Data from 1,543 patients were retrospectively analyzed, and their general conditions, including sex, age, diabetes duration, height, weight, systolic blood pressure (SBP), diastolic blood pressure (DBP), history of smoking and drinking, history of high blood pressure (HBP), history of diabetic retinopathy (DR), and history of diabetic nephropathy (DN) were recorded. The body mass index (BMI) was calculated as follows: BMI (kg/m^2^) = weight (kg) / body height^2^ (m^2^).

### 2.4 Laboratory biochemical indices

All participants fasted for more than 8 h, and the blood biochemical indices were measured the following morning of the next day, including C-reactive protein (CRP), glycosylated hemoglobin (HbA1c), fasting blood glucose (FBG), triglyceride (TG), total cholesterol (TC), high-density lipoprotein cholesterol (HDL-C), low-density lipoprotein cholesterol (LDL-C), serum creatinine (sCr), uric acid (UA), aspartate aminotransferase (AST), alanine aminotransferase (ALT), and albumin (ALB). The glomerular filtration rate (eGFR) was calculated according to the sCr. Postprandial blood glucose (PBG) levels were measured 2 h after the first meal bite. Biochemical indices were tested using a Beckman Coulter U5811 (USA). HbA1c was determined by high-performance liquid chromatography using the detection instrument model of the Japan Dongcao G8 glycosylated hemoglobin analyzer.

The glomerular filtration rate (eGFR) was calculated based on the sCr values of the study participants as follows:

Male:

sCr ≤ 0.9 mg/dl: eGFR CKD-EPI-ASIA = 141 × (sCr/0.9) – 0.411 × 0.993 age × 1.057
sCr > 0.9 mg/dl: eGFR CKD-EPI-ASIA = 141 × (sCr/0.9) – 1.209 × 0.993 age × 1.057

Female:

sCr ≤ 0.7 mg/dl: eGFR CKD-EPI-ASIA = 141 × (sCr/0.7) – 0.329 × 0.993 age × 1.049
sCr > 0.7 mg/dl: eGFR CKD-EPI-ASIA = 141 × (sCr/0.7) – 1.209 × 0.993 age × 1.049

### 2.5 EVA diagnosis

We use a BP-203 III automatic atherosclerosis analyzer (Omron, Tokyo, Japan) to measure ankle-brachial index (ABI) and PWV values. The left and right ABI were calculated as the systolic ankle pressure divided by the systolic brachial pressure. ABI and PWV values were measured and assessed by the same group of physicians in the Department of Endocrinology to avoid potential interexaminer differences.

The diagnostic criterion for EVA is the condition in which the PWV value after adjusting for age is two standard deviations higher than the normal reference value of the healthy population (2). Patients were divided into non-EVA and EVA groups based on PWV values.

### 2.6 Statistical methods

Data analyses were performed using the SPSS software (version 21.0; SPSS Inc., Chicago, IL, USA). The *t*-test and Wilcoxon test were used to compare data between the two groups for normally and non-normally distributed data, respectively. The χ^2^ test was used for comparisons between the two groups. Unconditional multivariable logistic regression analysis was used to calculate odds ratios (ORs) and corresponding 95% confidence intervals (95% CIs). Differences were considered statistically significant at *p* < 0.05. We used multiple imputations to supplement the missing values of the variables.

We use packages R (https://www.R-project.org) and EmpowerStats (https://www.empowerstats.com, X&Y Solutions, Inc., Boston, MA) to establish the nomogram. Receiver operating characteristic (ROC) curves were used to evaluate the discriminatory ability of the nomogram, and the area under the ROC curve (AUC) was calculated using 500 bootstrap resamplings.

## 3 Results

### 3.1 Comparison of general conditions and biochemical indexes between the two groups

The incidence of EVA in T2DM patients was 51.33%. Compared to the non-EVA group, the EVA group had a significantly higher incidence of HBP (χ^2^ = 55.29, *p* < 0.05). Compared with the non-EVA group, the patients in the EVA group were older and had a longer diabetes duration, with statistically significant differences (*t* = −6.57, *p* < 0.05; *t* = −10.61, *p* < 0.05). Compared with the non-EVA group, patients in the EVA group had significantly higher PBG levels and lower eGFR levels, with statistically significant differences (*t* = −2.00, *p* < 0.05; *t* = 5.22, *p* < 0.05). PWV levels in the EVA group were higher than those in the non-EVA group, and ABI was significantly lower in the EVA group than in the non-EVA group (*t* = −27.29, *p* < 0.05; *t* = 3.87, *p* < 0.05). The CIMT levels in the EVA group were higher than those in the non-EVA group; however, the difference was not statistically significant (*p* > 0.05). The incidence of DR and DN in the EVA group was significantly higher than that in the non-EVA group (χ^2^ = 44.28, *p* < 0.05; χ^2^ = 96.30, *p* < 0.05, respectively). The proportion of smokers in the EVA group was significantly higher than that in the non-EVA group (χ^2^ = 21.02, *p* < 0.05). There were no significant differences in sex, drinking status, BMI, FBG, HbA1c, AST, ALT, ALB, TC, TG, LDL-C, HDL-C, UA levels, and medications between the two groups (*p* > 0.05; Table 1).

**Table 1:** Comparison of general conditions and biochemical indexes between the two groups.

**Index**	**Non-EVA group (*n* = 751)**	**EVA group (*n* = 792)**	***t* (χ^2^)**	***p-*value**
Age (years)	53.39 ± 13.55	57.76 ± 12.67	−6.57	< 0.001
**Sex**				
Male	476 (63.38%)	507 (64.02%)	0.07	0.80
Female	275 (36.62%)	285 (35.98%)		
Duration (years)	7.71 ± 7.83	11.74 ± 7.61	−10.61	< 0.001
BMI (kg/m^2^)	25.71 ± 2.83	25.71 ± 3.46	0.01	0.99
SBP (mmHg)	127.74 ± 17.99	137.068 ± 16.86	−10.84	< 0.001
DBP (mmHg)	77.94 ± 11.51	80.84 ± 10.73	−5.15	< 0.001
HBP (%)	219 (29.16%)	377 (47.60%)	55.29	< 0.001
Smoking (%)	125 (16.64%)	263 (33.21%)	21.02	< 0.001
Drinking (%)	112 (14.91%)	122 (15.40%)	0.82	0.77
CRP (mg/L)	5.64 ± 1.32	6.05 ± 0.98	−0.99	0.32
FBG	8.97 ± 3.31	9.15 ± 3.39	−1.05	0.29
PBG	12.58 ± 5.50	13.11 ± 4.57	−2.00	0.01
TC (mmol/L)	4.42 ± 1.17	4.32 ± 1.22	1.64	0.10
TG (mmol/L)	2.03 ± 1.38	1.96 ± 1.39	1.07	0.29
LDL-C (mmol/L)	2.57 ± 0.94	2.54 ± 0.91	0.45	0.65
HDL-C (mmol/L)	1.01 ± 0.30	1.03 ± 0.29	−1.17	0.24
eGFR (ml/min/1.73 m^2^)	100.26 ± 19.59	95.03 ± 19.15	5.22	< 0.001
UA (mmol/L)	344.50 ± 97.83	349.17 ± 89.70	−0.96	0.33
AST	23.42 ± 26.08	22.53 ± 23.24	1.10	0.25
ALT	26.65 ± 19.67	24.88 ± 15.37	1.83	0.19
ALB	44.73 ± 29.67	41.53 ± 29.02	1.36	0.17
HbA1c (%)	8.78 ± 1.97	8.83 ± 2.03	−0.49	0.62
PWV	1,401.15 ± 398.01	1,846.78 ± 380.00	−27.29	< 0.001
ABI	1.15 ± 0.12	1.13 ± 0.13	3.87	< 0.001
CIMT	1.09 ± 0.54	1.18 ± 0.54	−1.77	0.08
**Complications**				
DN (%)	123 (16.38%)	244 (30.81%)	44.28	< 0.001
DR (%)	97 (12.92%)	271 (34.22%)	96.30	< 0.001
**Medicine**				
Metformin	491 (65.38%)	502 (63.38%)	4.32	0.65
Sulfonylureas	103 (13.72%)	114 (14.39%)	3.23	0.78
Insulin	234 (31.16%)	297 (37.50%)	5.44	0.43
SGLT-2 inhibitors	372 (49.53%)	352 (44.44%)	4.83	0.50
GLP-1 receptor agonists	293 (39.01%)	297 (37.5%)	6.32	0.16
Thiazolidinedione	79 (10.52%)	101 (12.75%)	4.71	0.38

BMI, body mass index; SBP, systolic blood pressure; DBP, diastolic blood pressure; HBP, high blood pressure; CRP, C-reactive protein; FBG, fasting blood glucose; PBG, postprandial blood glucose; HbA1c, glycosylated hemoglobin; TC, total cholesterol; TG, triglyceride; LDL-C, low-density lipoprotein cholesterol; HDL-C, high-density lipoprotein cholesterol; AST, aspartate aminotransferase; ALT, alanine aminotransferase; ALB, albumin; UA, uric acid; eGFR, estimated glomerular filtration rate; PWV, pulse wave velocity; ABI, ankle-brachial index; CIMT, carotid intima-media thickness; DN, diabetic nephropathy; DR, diabetic retinopathy; SGLT-2, sodium-dependent glucose transporters 2; GLP-1, glucagon-like peptide-1.

### 3.2 Multivariable logistic regression analysis of risk factors for EVA occurrence in T2DM patients

Multivariate logistic regression analysis was performed with EVA as the dependent variable and baseline characteristics (age, duration, BMI, HBP, smoking, HbA1c, TC, TG, DN, and DR) as independent variables. The risk factors for EVA in male included: longer diabetic duration (OR = 1.09 95 CI% 1.06–1.11), HBP (OR = 2.06, 95 CI% 1.51–2.81), smoking (OR = 1.96 95 CI% 1.17–3.27), DN (OR = 1.60 95 CI% 1.10–2.32), and DR (OR = 2.93 95 CI% 2.00–4.29).

Multivariate logistic regression analysis was performed with EVA as the dependent variable and baseline characteristics (age, duration, BMI, smoking, HBP, HbA1c, TC, TG, DN, and DR) as independent variables. The risk factors for EVA in female included longer diabetic duration (OR = 1.04 95 CI% 1.01–1.07), smoking (OR = 2.02, 95 CI% 1.13, 3.59), HBP (OR = 1.91, 95 CI% 1.22–2.79), DN (OR = 1.61 95 CI% 1.02–2.52), and DR (OR = 3.61 95 CI% 2.24–5.74; Table 2).

**Table 2:** Univariate logistic regression analysis of EVA.

**Index**	**Crude OR**	**95% CI**	***p-*value**	**Adjust OR**	**95% CI**	***p-*value**
**Male**				
Age	1.03	1.02, 1.04	< 0.001	1.01	1.00, 1.02	0.20
Duration	1.06	1.03, 1.09	< 0.001	1.09	1.06, 1.11	< 0.001
HBP	2.51	1.81, 3.48	< 0.001	2.06	1.51, 2.81	< 0.001
BMI	1.00	0.97, 1.04	0.79	1.03	0.99, 1.06	0.14
Smoking	2.51	1.81, 3.48	< 0.001	1.96	1.17, 3.27	< 0.001
TC	0.86	0.77, 0.95	< 0.001	0.94	0.84, 1.06	0.30
TG	0.91	0.83, 0.99	< 0.001	0.96	0.87, 1.06	0.44
HbA1c	0.98	0.91, 1.06	0.65	1.01	0.94, 1.08	0.83
DR	3.71	2.29, 4.39	< 0.001	2.93	2.00, 4.29	< 0.001
DN	2.32	1.69, 3.18	< 0.001	1.60	1.10, 2.32	0.01
**Female**				
Age	0.99	0.96, 1.01	0.36	1.01	0.95, 1.06	0.43
Duration	1.07	1.04, 1.09	< 0.001	1.04	1.01, 1.07	< 0.001
HBP	2.20	1.55, 3.12	< 0.001	1.91	1.22, 2.79	< 0.001
BMI	0.99	0.95, 1.04	0.64	1.00	0.95, 1.05	0.88
Smoking	2.47	1.72, 3.57	< 0.001	2.02	1.13, 3.59	< 0.001
TC	1.05	0.88, 1.25	0.58	1.14	0.98, 1.32	0.09
TG	1.02	0.86, 1.21	0.82	1.10	0.97, 1.26	0.15
HbA1c	1.07	0.98, 1.17	0.11	1.04	0.94, 1.14	0.46
DR	4.11	2.68, 6.31	< 0.001	3.61	2.24, 5.74	< 0.001
DN	2.20	1.49, 3.25	< 0.001	1.61	1.02, 2.52	0.004

BMI, body mass index; HBP, high blood pressure; HbA1c, glycosylated hemoglobin; TC, total cholesterol; TG, triglyceride; DN, diabetic nephropathy; DR, diabetic retinopathy.

### 3.3 Predictive nomograms model for EVA and predictive accuracy of nomograms

**Figure 1:**
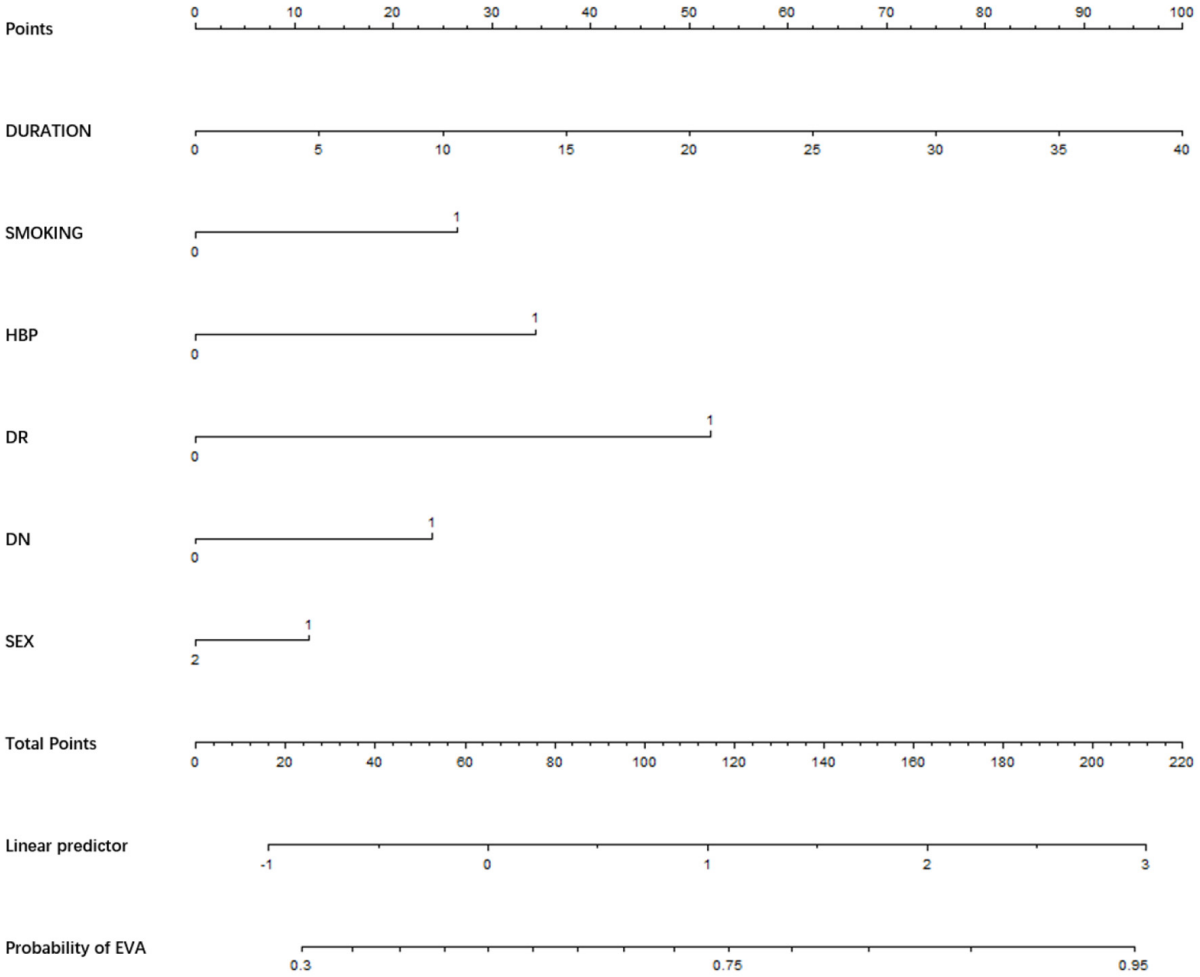
Nomogram for EVA in patients with T2DM. Instructions: to estimate an individual's EVA risk for female with T2DM, his/her value on each variable axis. Draw a verticle line from that value to the top that Points scale for determining how many points are assigned by that variable valule. Then, the points from each variable value are summed. Locate the sum on the Total Points sacle and vertically project in onto the bottom axis, then obtaining a personalized EVA risk of T2DM.

**Figure 2:**
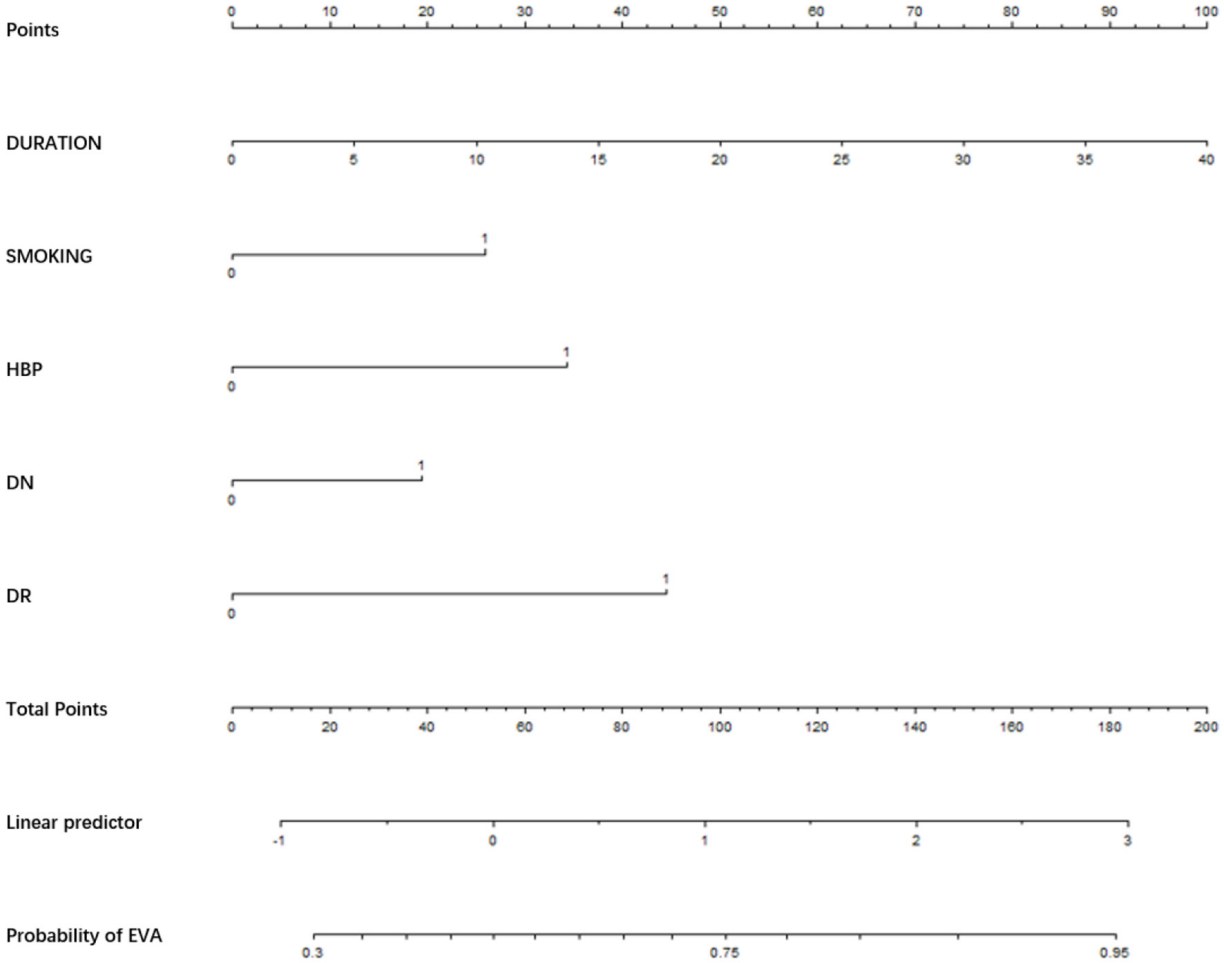
Nomogram for EVA in male with T2DM. Instructions: to estimate an individual's EVA risk for male with T2DM, his value on each variable axis. Draw a verticle line from that value to the top that Points scale for determining how many points are assigned by that variable valule. Then, the points from each variable value are summed. Locate the sum on the Total Points sacle and vertically project in onto the bottom axis, then obtaining a personalized EVA risk of T2DM.

**Figure 3:**
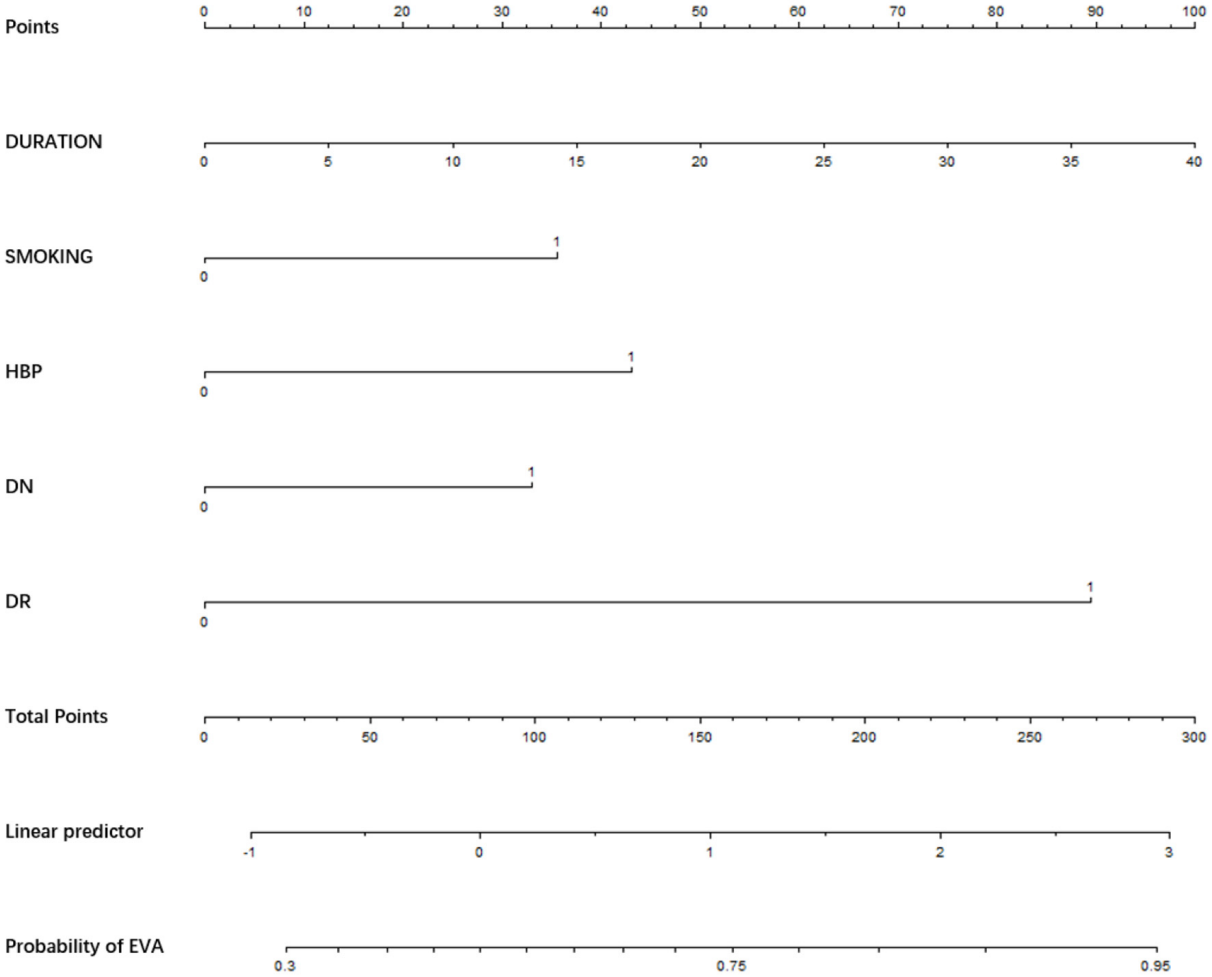
Nomogram for EVA in female with T2DM. Instructions: to estimate an individual's EVA risk for female with T2DM, her value on each variable axis. Draw a verticle line from that value to the top that Points scale for determining how many points are assigned by that variable valule. Then, the points from each variable value are summed. Locate the sum on the Total Points sacle and vertically project in onto the bottom axis, then obtaining a personalized EVA risk of T2DM.

We used the risk factors for EVA obtained from the multivariable logistic regression analysis as variables in the prediction nomogram of EVA. Nomograms for evaluating the risk of EVA in males and females were developed for T2DM patients based on risk factors identified by multivariable logistic regression analysis (Figures 1–3).

**Figure 4:**
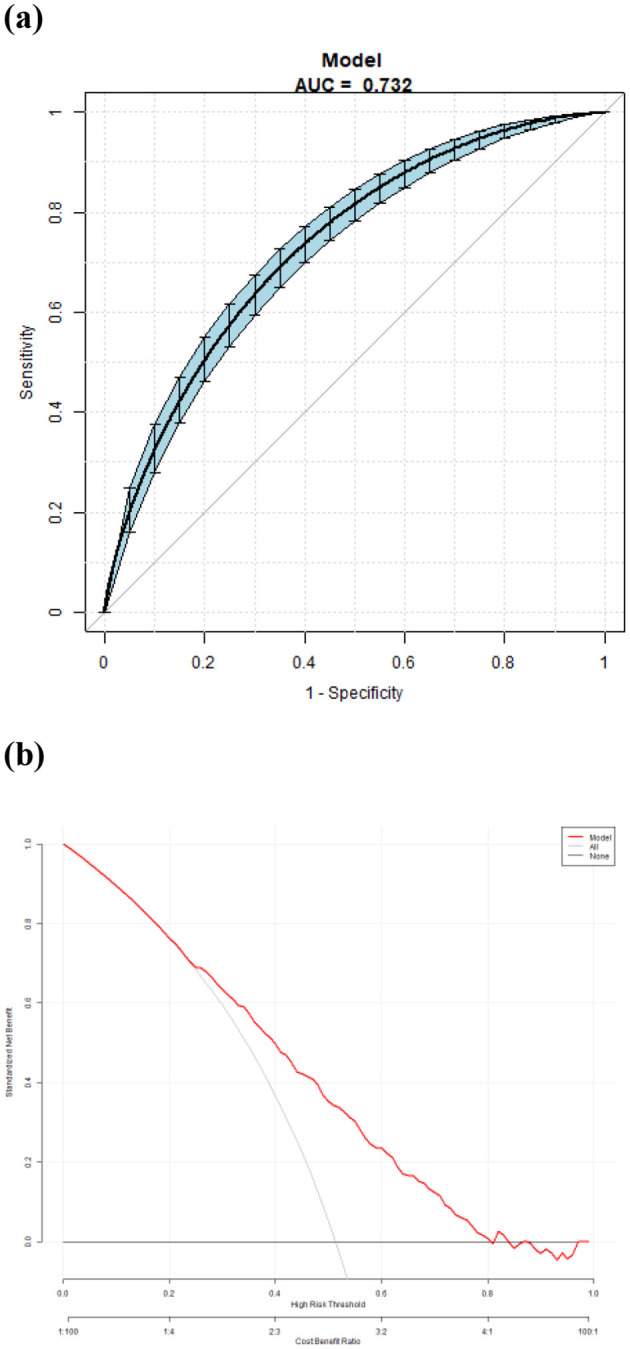
**(a)** Receiver operating characteristic (ROC) curves for the accuracy of the EVA nomogram with T2DM. The AUC of the Model is 0.732. The overall predictive accuracy of the nomogram for EVA was 68.374%, and the specificity and sensitivity were 69.07% and 68.37%, respectively. **(b)** Decision curve analysis (DCA) for the EVA nomogram with T2DM.

**Figure 5:**
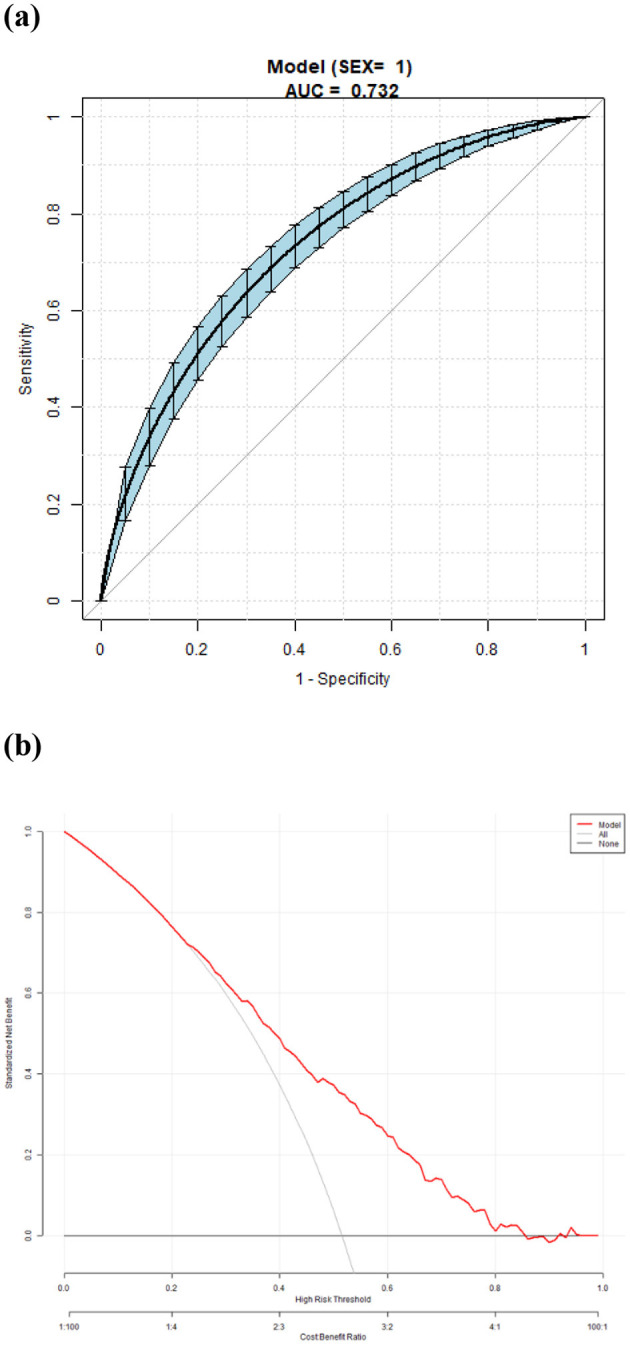
**(a)** Receiver operating characteristic (ROC) curves for the accuracy of the EVA nomogram in male with T2DM. The AUC of the Model is 0.732. The overall predictive accuracy of the nomogram for EVA was 67.85%, and the specificity and sensitivity were 73.74% and 62.33%, respectively. **(b)** Decision curve analysis (DCA) for the EVA nomogram in male with T2DM.

**Figure 6:**
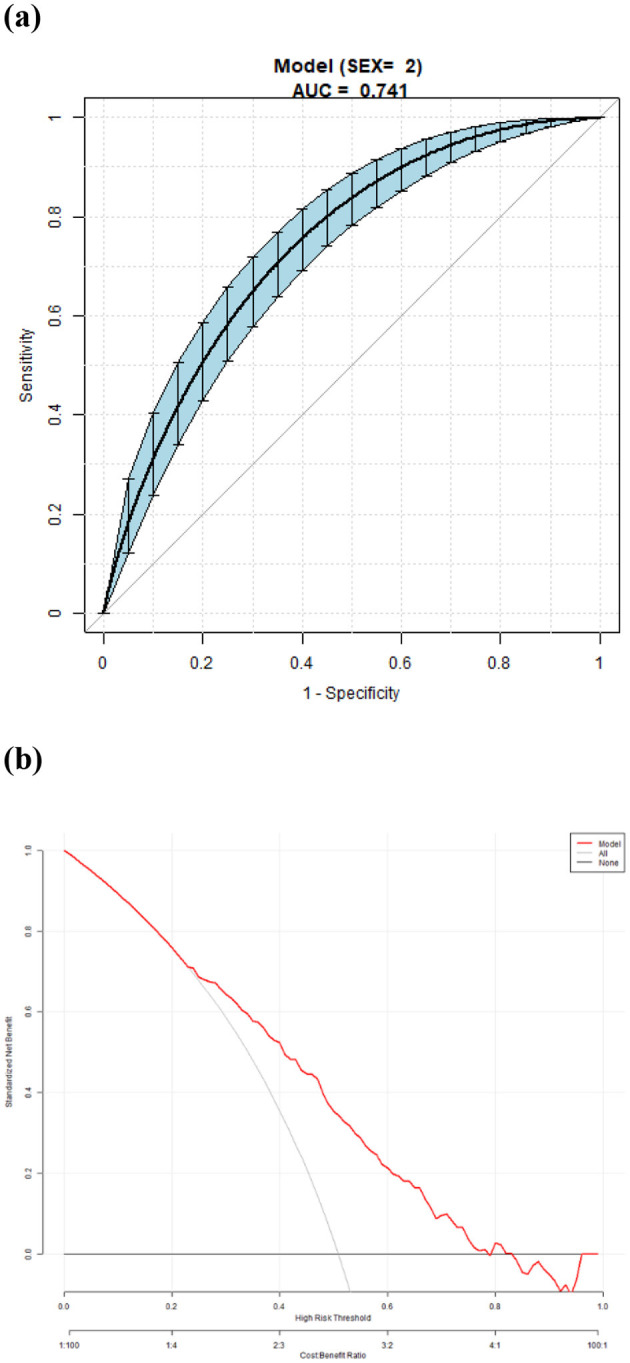
**(a)** Receiver operating characteristic (ROC) curves for the accuracy of the EVA nomogram in female with T2DM. The AUC of the Model is 0.741. The overall predictive accuracy of the nomogram for EVA was 69.29%, and the specificity and sensitivity were 66.55% and 71.93%, respectively. **(b)** Decision curve analysis (DCA) for the EVA nomogram in female with T2DM.

The area under the curve (AUC) of the model was 0.732. The overall predictive accuracy of the nomogram for EVA was 68.374%, and the specificity and sensitivity were 69.07% and 68.37%, respectively. The AUC of the nomogram for EVA in male is 0.73 and in female is 0.74. The overall predictive accuracy of the nomogram for EVA in men was 67.85%, with a specificity and sensitivity of 73.74% and 62.33%, respectively. The overall predictive accuracy of the nomogram for EVA in females was 69.29%, with a specificity and sensitivity of 66.55% and 71.93%, respectively (Figures 4–6).

## 4 Discussion

Individuals with T2DM are at a higher risk of cardiovascular events than those in the general population. Several studies have investigated the association between EVA and T2DM (8–11). These studies have consistently demonstrated that T2DM patients have a higher prevalence and severity of EVA than those without T2DM. Several studies have attempted to develop risk prediction models for EVA in T2DM patients. These models aim to identify high-risk patients and provide clinicians with tools for early interventions (12, 13). However, the above research results only identified the risk factors for EVA and did not establish a simple and practical prediction model for each individual. The mechanism of EVA in patients with T2DM includes the following aspects: (1) inflammatory reaction (14): the balance of advanced glycation end products (AGEs) in T2DM patients is broken and accumulated in large quantities, which can block the insulin pathway and promote inflammatory reactions (15). Simultaneously, elastin rupture, collagen deposition, vasodilation, and inner diameter of arterial muscle cells can lead to a decrease in vascular elasticity and hardness, while inflammatory reactions and oxidative stress can cause autophagy to hinder angiogenesis, leading to aggravation of vascular aging (16). (2) Inhibited autophagy pathway (17). Autophagy is one of the main pathways to promote the clearance of AGEs, and blocking this pathway can lead to the accumulation of AGEs and the production of inflammatory cytokines, which can lead to endothelial injury of endothelial functional cells in T2DM patients and aggravate vascular aging (18). (3) Ang II: T2DM can promote the use of Ang II in muscle cells to upregulate the expression of DNA methyltransferase, leading to thickening of the blood vessel wall (19). Although both mechanistic and clinical studies have confirmed that T2DM patients are prone to EVA, there is still a lack of a simple and practical clinical model at home and abroad to demonstrate the prediction of EVA occurrence probability in T2DM patients. There is a need for a specific risk prediction model for EVA in T2DM patients. This study aimed to develop a nomogram-based risk prediction model for EVA in T2DM patients.

In our study, diabetes duration was identified as a significant predictor. This suggests that the longer an individual lives with T2DM, the higher the risk of EVA. This finding highlights the importance of early diagnosis and intervention in T2DM patients to prevent or delay the occurrence of EVA. Additionally, HBP was identified as an important predictor of EVA in patients with T2DM. Elevated blood pressure is a known risk factor for cardiovascular disease and has been associated with vascular aging. Our study emphasizes the need for monitoring and managing blood pressure in T2DM patients to identify the risk of EVA. In our study, smoking was a risk factor for EVA in both male and female patients; smoking increased free radicals (such as peroxides) in the body, thereby decreasing vascular endothelial diastolic function, resulting in arterial contraction. This promotes arterial stenosis, accelerates arteriosclerosis, aggravates limb ischemia, and ultimately leads to EVA (20). DN and DR are the most common microvascular complications. Proteinuria is an independent risk factor for diabetes and cardiovascular mortality. Systemic endothelial dysfunction is a common underlying factor of DN and vascular aging (21). Our study revealed an association between DN and EVA. The risk of EVA was 1.60 times higher in males and 1.61 times higher in females. Therefore, it is recommended that patients with T2DM actively monitor their urinary microalbumin levels. If the levels of urinary microalbumin are found to be abnormal, it is necessary to regularly check the vascular condition to take active care and obtain preventive treatment. Several studies have shown a strong association between DR and macrovascular diseases, such as cardiovascular disease in T2DM patients (22, 23). Multivariable logistic regression analysis showed that DR was an independent risk factor for EVA in both men and women, with OR of 2.93 and 3.91, respectively. This may be because the pathogenesis of these two diseases shares common risk factors (24). There was no significant difference in BMI between the EVA and non-EVA groups in our study. BMI was not an independent factor influencing the EVA. Similar results have been found in different populations in China and Germany (25–27). Some studies suggest that a high BMI may be a protective factor for arteriosclerosis in males aged 35–55 years (27). This finding may be related to the obesity paradox. Obesity has many adverse effects on the body, but it also has some benefits. Although obesity may be an important factor in arterial remodeling, leading to changes in hemodynamics and arterial structure affecting normal vascular function, the vascular endothelial function of obese individuals may still be normal (26). Simultaneously, obese patients have higher nutritional reserves to cope with acute stress events and metabolic needs. Adipose tissue produces beneficial cytokines. Individuals with insulin-induced obesity have good metabolic characteristics. The interaction between these risks and benefits may lead to uncertainty regarding the correlation between BMI and vascular aging. Therefore, the association between obesity and vascular aging remains to be elucidated. Whether vascular aging is one of the decisive mechanisms of the obesity paradox remains to be confirmed in future multicenter prospective studies. In addition, we did not observe a significant correlation between dyslipidemia and poor blood glucose control with vascular aging. Our study participants were outpatients and inpatients with T2DM whose blood glucose and lipid levels were generally poorly controlled, which may have led to bias in the study results and did not represent whether there was a close relationship between blood glucose and lipid levels with EVA. Currently, as a widely used statistical tool in clinical practice, the nomogram can directly calculate and display the contribution of various variables to the results, which may help evaluate their importance and impact and can also be used for the prediction of many diseases in clinical practice (24). The nomogram is a graphical representation that assigns a score to each predictor based on its contribution to EVA risk. The scores for each predictor were summed to obtain a total score that could be used to estimate an individual's risk of developing EVA. In this study, we developed a nomogram-based risk prediction model for EVA in patients with T2DM. In our study, the results of the risk prediction model demonstrated that diabetic duration, HBP, smoking, DN and DR were important predictors for EVA in patients with T2DM. Nomograms were constructed based on these predictors. To validate the nomogram-based risk prediction model, the performance of the model was assessed using various statistical metrics, such as sensitivity, specificity, accuracy and ROC. The results showed that the nomogram-based risk prediction model achieved a high AUC of 0.73 for male and 0.74 for female. The sensitivity and specificity of the model were 62.33% and 73.74%, respectively, in males and 71.93% and 66.55%, respectively, in females. Therefore, nomograms with AUC greater than 0.7 have good predictive ability. There is no predictive model for EVA in T2DM patients in the past. This study is also the first to use the risk factors of EVA as a predictive index, which is presented through a simple-to-use nomogram and has a certain degree of accuracy. This can provide a more comprehensive clinical approach for the early detection and intervention of EVA in T2DM patients.

This study had some limitations. First, this was a single-center cross-sectional study, and a prospective study with a larger sample size should be conducted at multiple centers in the future. Second, we used an internal cohort for validation. In the future, external cohort validation may be used to better reveal the predictive ability of the nomogram model. Finally, to better reveal the risk factors for EVA in T2DM patients, we will include more T2DM participants with stable blood glucose levels to avoid bias caused by blood glucose and blood lipid levels. In addition, this was a retrospective study, and we included as many factors as possible that had already been obtained. We also supplemented the medication history and inflammatory indicators; however, there were no data on physical activity and dietary habits. In future studies, we will supplement relevant data and explore additional risk factors and markers to improve the predictive performance of the model.

## 5 Conclusion

In conclusion, this study found that the incidence of EVA in T2DM patients was 51.33%. In both men and women, the risk factors of EVA in patients with T2DM included age, duration, BMI, HBP, smoking, HbA1c, TC, TG, DN, and DR.

The nomogram-based risk prediction models exhibited good discriminative ability and calibration performance, indicating the reliability of estimating the risk of EVA in T2DM patients. These models provide clinicians with a reliable tool for identifying high-risk patients and implementing early interventions to reduce the burden of cardiovascular diseases.

## Data Availability

The data analyzed in this study is subject to the following licenses/restrictions: The datasets generated during this study are available from the corresponding author upon reasonable request. Requests to access these datasets should be directed to: zhaoxin2012@aliyun.com.
